# SHD reviewers January 2025–December 2025

**DOI:** 10.1093/skinhd/vzag016

**Published:** 2026-03-31

Successful and reliable publishing is dependent on expert, objective and timely peer review. The editorial team greatly appreciates the goodwill, time and effort that goes into writing these reviews and I would like to thank all those who reviewed for SHD last year.

Abbaspour, Elahe

Abbott, Rachel

Abdel Azim, Amira

Abdel Naser, Mohamed Badawy

Abdelmaksoud, Ayman

Abu El-Hamd, Mohammed

Adamson, Adewole

Affleck, Andrew

Aggarwal, Divya

Agut-Busquet, Eugenia

Ahn, Ga Ram

Akasaka, Eijiro

Akhras, Victoria

Alaibac, Mauro

Alassaf, Jihad

Albela, Henrietta

Alfonso, Jose Hernan

Ali Khan, Imran

Allsop, Daniel

Al-Muriesh, Maher

Alsharabati, Muntasser

Álvarez-Salafranca, Marcial

Andersen, Klaus

Andersen, Monica

Ansai, Osamu

Aristizabal, Miguel

Aromolo, Italo Francesco

Ascott, Anna

Ashique, Karalikkattil

Asif, Aqua

Assaf, Chalid

Attia, Enas

Audet, Samuel

Aung, Tim

Babker, Asaad M.A.

Babuna Kobaner, Goncagul

Balieva, Flora

Baran, Anna

Bashyam, Arjun

Baskurt, Defne

Bassas-Vila, Julio

Bataille, Veronique

Bhattacharya, Shatanik

Bighetti, Stefano

Bisoyi, Diptiranjani

Boch, Katharina

Boey, Justin Jia Jun

Bollemeijer, Juliette

Boniotto, Michele

Boyar, Vita

Bradshaw, Sarah

Bristow, Ivan

Brocklehurst, Jonathan

Bueno, Juan

Burden, David

Butt, Sundas

Caballero-Linares, Cristian Fernando

Calzavara-Pinton, Pier Giacomo

Cao, Taige

Cappilli, Simone

Cardenas-De La Garza, Jesus Alberto

Carmichael, Andrew

Carroccio, Antonio

Carroll, Liam

Castro-Martin, Julia

Chen, Yi-Ju

Chernyshov, Pavel

Cho, Yung-Tsu

Choi, Ellie

Cole-Adeife, Olufolakemi

Corona, Maria Fernanda

Corrà, Alberto

Daneshpazhooh, Maryam

Das, Kinnor

Daveluy, Steven

De Almeida, H.

De Andrea, Marco

De Graaf, Marlies

De Troya-Martin, Magdalena

Dear, Katherine

Di Guardo, Antonio

Diaz Calvillo, Pablo

Diffey, Brian

Dingle, Rebecca

Docampo-Simón, Alexandre

Dolianitis, Con

Donovan, Jeffrey

Du Harpar, Beibei

Dubash, Faisal

Dubois, Anna

Dyson, Sarah

Eecen, Christina

Egawa, Gyohei

Eid, Amira

Emelianov, Vladimir

Emmanuel, Thanos

Esdaile, Ben

Faita, Francesco

Fang, Sheng

Faridzadeh, Arezoo

Farista, Arshi

Fatemian, Hossein

Fearfield, Louise

Feldman, Steven

Ferreira, João

Firnhaber, Jonathon M.

Firooz, Alireza

Fityan, Adam

Fujiwara, Hiroshi

Fuller, Lucinda

Gaeta, Aurora

Galli, Andrea

Galvan Casas, Cristina

García-Moronta, Carmen

Gawkrodger, David

George, Anju

George, Susannah

Ger, Tzong Yun

Gerbens, Louise

Girma, Abayeneh

Godor, Dorottya

Goebeler, Matthias

Gogineni, Ranga

Gohar, Amr

Goldenberg, Michael

Gönülal, Melis

Goodwill, Joseph

Gordon, Kristiana

Gostynski, Antoni

Guarro, Giuseppe

Guo, Zongsheng

Hagino, Teppei

Hampton, Philip

Haneke, Eckart

Has, Cristina

Hasan Pour, Bahareh

Hasan, Samar

Hasan, Shamimul

Heelan, Kara

Hegde, Shibani

Hieta, Niina

Honan, Alison

Honnor, Kate

Howell, Michael

Hsu, Chao-Kai

Hsu, Ming-Long

Hughes, Zara

Hussain, Walayat

Ibbotson, Sally

Iino, Noriaki

Inamadar, Arun

Ingram, John

Jaiswal, Sunil

Jebril, William

Jerkovic Gulin, Sandra

Jiang, Hao

Joghataei, Mohammad Taghi

Kamminga, Nadia

Kanelleas, Antonios

Kanitakis, Jean

Karampinis, Emmanouil

Karponis, Dimitrios

Katoch, Saloni

Katoulis, Alexandros

Kaya, Gökhan

Kayaaltı, Ahmet

Keith, Paul

Kentley, Jonathan

Khan, Farishta

Khan, Sidra

Khunger, Niti

King Stokes, Natalie

Kinoshita-Ise, Misaki

Kinouchi, Motoshi

Kiridaran, Vaishali

Koga, Hiroshi

Kokkonen, Nina

Konda, Sharath Chandra

Kono, Michihiro

Kostik, Mikhail M.

Kotb, Iman

Kounidas, Georgios

Kulkarni, Rajan

Kulkarni, Sandeep

Kurtansky, Nicholas

Labib, Shery

Laheru, Dhruv

Lai, Michela

Lallas, Konstantinos

Langan, Ewan

Lapin, Agnieszka

Laws, Philip

Lawson, Inga

Lawton, Sandra

Leducq, Sophie

Li, Si-Zhe

Li, Wei

Lim, Zhi

Lin, Jianjian

Lin, Kuo-Hsi

Lio, Peter

Liu-Smith, Feng

Lockwood, Katie

Long, Valencia

Lu, Chan

Lu, Chun-Wei

Maglie, Roberto

Mahmood, Muhammad

Mahmoudi, Hamidreza

Maione, Vincenzo

Manolache, Liana

Martins, Giselle

Mathews, Irene

Matin, Rubeta

Meijer, Joost

Metin, Mahmut Sami

Millette, Dijon

Millington, George

Misery, Laurent

Mitchell, Charles

Miteva, Mariya

Miyachi, Hideaki

Miyagaki, Tomomitsu

Miyashiro, Denis

Mohandas, Padma

Mohapatra, Liza

Moiz, Haseeb

Molina-Leyva, Alejandro

Molloy, Kevin

Montero-Vilchez, Trinidad

Morales-Sánchez, Martha Alejandra

Morrow, Sarah

Muir, Jim

Mun, Andrey

Munir, Tahimina

Murphy, Ruth

Murray, Gregg

Murrell, Dedee

Muthusamy, Annamalai

Nair, Aswin M.

Nallathamby, Kasthuri

Nambudiri, Vinod

Narang, Tarun

Nazzaro, Gianluca

Ng, Chau Yee

Nielsen-Scott, Anna

Nikookam, Yasmin

Nomura, Toshifumi

Nylander, Elisabet

Ogboli, Malobi

Ohn, Jungyoon

Ortiz-Álvarez, Juan

Oyewole, Olufemi O.

Palanivel, Janani

Papas, Athanasios

Papp, Kim

Pasquali, Paola

Patel, Mitesh

Patel, Tejesh

Paudel, Supriya

Paudel, Vikash

Peng, Hsien-Li Peter

Perandones-González, Héctor

Persechino, Flavia

Peters, Eva

Petrof, Gabriela

Pham, James

Pincelli, Carlo

Porebski, Grzegorz

Poudel, Pratima

Pudasaini, Prajwal

Rajabi, Fatemeh

Ramessur, Ravi

Rapiti, Nadine

Ray, Anish

Reay, William

Recke, Andreas

Reich, Adam

Reihanian, Zoheir

Reis, Moritz

Rittie, Laure

Rodriguez-Sanna, Andrea

Rolls, Sophie

Rosen, Cheryl

Ruiz-Villanueva, Alejandra

Rungapiromnan, Watcharee

Russo, Filomena

Sanabria De La Torre, Raquel

Sanader Vucemilovic, Ana

Sanchez-Diaz, Manuel

Sarkany, Robert

Schmitt-Egenolf, Marcus

Scope, Alon

Searle, Tamara

Sechi, Andrea

Senkal, Emine

Sepaskhah, Mozhdeh

Shah, Bibhant

Shah, Sandesh

Shrestha, Elisha

Shukla, Prakriti

Sil, Abheek

Singh, Jasleen

Smak Gregoor, Anna

Sodre, Camila

Solimani, Farzan

Song, Junhyuk

Soto-Moreno, Alberto

Spence, Colin

Starace, Michela

Stavrou, Christiana

Steijlen, Olivia

Svensson, Åke

Szadai, Leticia

Szczecinska, Weronika

Szpakow, Andriej

Takwale, Anita

Taleb, Eyal

Tan, Kathryn

Tan, Yingrou

Tang, Edwina

Tang, Yan

Tanweer, Tahreem

Temiz, Selami Aykut

Thiem, Alexander

Thierauf, Julia

Todorova, Antonia

Toni, Emmanuel

Torchia, Daniele

Tsao, Hensin

Tseng, Han-Chi

Tso, Simon

Turner, Madeleine

Ufodiama, Chiedu

Urena-Paniego, Clara Amanda

Vahabi, Seyed Mohammad

Vahidnezhad, Hassan

Van Beek, Nina

Van Doorn, Remco

Van Huijstee, Johanna

Vorobyev, Artem

Wainman, Hannah

Walsh, Sarah

Wan, Hong

Wang, Hui

Wang, Tao

Washio, Ken

Weller, Richard

Widhiati, Suci

William, Basem M.

Winn, Remus

Wozniak, Katarzyna

Wu, Wei-Hsin

Wu, Weiwei

Yancheva, Nina

Yang, Wei-Hua

Yorulmaz, Ahu

Zaouak, Anissa

Zengarini, Corrado

Zhang, Jing-Zhan

Zhang, Wei

Zhang, Xiaoting

Zheng, David

Zhifang, Zhai

Zhuang, Kaiwen

Zou, Qin

Zouboulis, Christos

Zwiener, Ricardo

## Author contributions

John Caulfield (Writing—original draft [lead])

